# The role of oxidative stress, tumor and inflammatory markers in colorectal cancer patients: A one-year follow-up study

**DOI:** 10.1016/j.redox.2023.102662

**Published:** 2023-03-08

**Authors:** Delia Acevedo-León, Segundo Ángel Gómez-Abril, Pablo Sanz-García, Nuria Estañ-Capell, Celia Bañuls, Guillermo Sáez

**Affiliations:** a Servicio de Análisis Clínicos, Hospital Universitario Dr. Peset-FISABIO, 46017, Valencia, Spain; b Servicio de Cirugía General y Aparato Digestivo, Hospital Universitario Dr. Peset-FISABIO, 46017, Valencia, Spain; c Servicio de Endocrinología y Nutrición, Hospital Universitario Dr. Peset-FISABIO, 46017, Valencia, Spain; d Departamento de Bioquímica y Biología Molecular, Facultad de Medicina y Odontotología, Universidad de Valencia, 46010, Valencia, Spain

**Keywords:** Colorectal cancer, Oxidative stress, Catalase, Glutathione system, 8-oxodG, F2-isoprotanes, CRC, Colorectal cancer, CAT, Catalase, GSH, Reduced glutahione, GSSG, Oxidized glutathione, 8-oxodG, 8-oxo-7,8-dihydro-2'-deoxyguanosine, CRP, C-reactive protein, IL-6, Interleukin 6, CEA, Carcinoembryonic antigen, CA 19.9, Carbohydrate antigen 19.9, OS, Oxidative stress, F2-IsoPs, F2-Isoprostanes, ROS, Reactive oxygen species

## Abstract

Oxidative stress (OS) and inflammation are known to play an important role in colorectal cancer (CRC). This study analyzed tumor, inflammatory and OS markers in CRC patients and in a control group. In addition, the evolution of these markers was evaluated after one-year of follow-up treatment. This was a longitudinal and prospective, observational study in 80 CRC patients who were candidates for tumor resection surgery and/or chemo-radiotherapy treatment and a healthy control group (n = 60). Subsequently, catalase (CAT), reduced glutathione (GSH), oxidized glutathione (GSSG) and GSSG/GSH ratio in serum and 8-oxo-7,8-dihydro-2′-deoxyguanosine (8-oxodG) and F2-IsoProstanes (F2-IsoPs) in urine at 1, 6 and 12 months after treatment was analyzed. Tumor markers (CEA and CA 19.9), as well as inflammatory markers—leukocytes, neutrophils, neutrophil/lymphocyte (N/L) index, platelets, fibrinogen, C-reactive protein (CRP), and interleukin 6 (IL6)— were also analyzed. As expected, levels of CEA and CA 19.9 and markers of inflammation, except CRP, were significantly higher in CRC compared to the control group. Regarding OS markers, a decrease in CAT and GSH and an increase in GSSG, GSSG/GSH ratio, 8-oxodG and F2-IsoPs were found in CRC patients compared to healthy controls at baseline. After treatment, an improvement of their inflammation profile was accompanied by a progressive recovery of antioxidant enzyme activities and the decline of oxidative byproducts both in serum and urine. Based on the results obtained, we propose the assay of urinary 8-oxodG and F2-IsoPs, as well as serum CAT, GSH, GSSG as a marker for the evaluation of OS and the clinical follow-up of CRC patients.

## Highlights

• To evaluate urinary and serum OS markers for the clinical monitoring of CRC patients.
• An important increase of OS was observed in CRC when compared with controls.
• A recovery of antioxidants and a decline of OS markers were observed after treatment.

## 1 Introduction

Colorectal cancer (CRC) is one of the main causes of cancer-related death in industrialized countries, according to data from Globocan 2020 [1]. Several risk factors are associated with the onset and progression of this tumor, such as environmental and lifestyle factors, like dietary pattern and physical inactivity [[2], [3], [4]].

At low levels, reactive oxygen species (ROS) have physiological cell functions, however they are toxic at high levels [5]. The imbalance in the oxidant/antioxidant equilibrium creates a condition known as oxidative stress (OS). In this context, ROS can cause oxidative cell damage leading to DNA and protein modification and lipid peroxidation [6]. These harmful effects can be controlled by natural antioxidant defense mechanisms and damage repair systems and antioxidants such as catalase (CAT) and reduced glutathione (GSH) [7]. OS and inflammation are known to play an important role in chronic diseases including cancer [8] and, specifically, CRC. ROS are involved in the initiation and progression of CRC [9,10].

In a review of the literature, different studies on the association of various markers of OS and antioxidants with CRC were observed [9,[11], [12], [13]], but no studies on the evolution of these markers before and after treatment of CRC patients were found. Therefore, our objective was to study antioxidant and OS markers, together with tumor and inflammatory markers used in clinical practice for the diagnosis of CRC, in order to check whether OS markers could be useful to improve the diagnosis and follow-up of CRC patients.

## 2 Material and methods

### 2.1 Study design

**Fig. 1:**
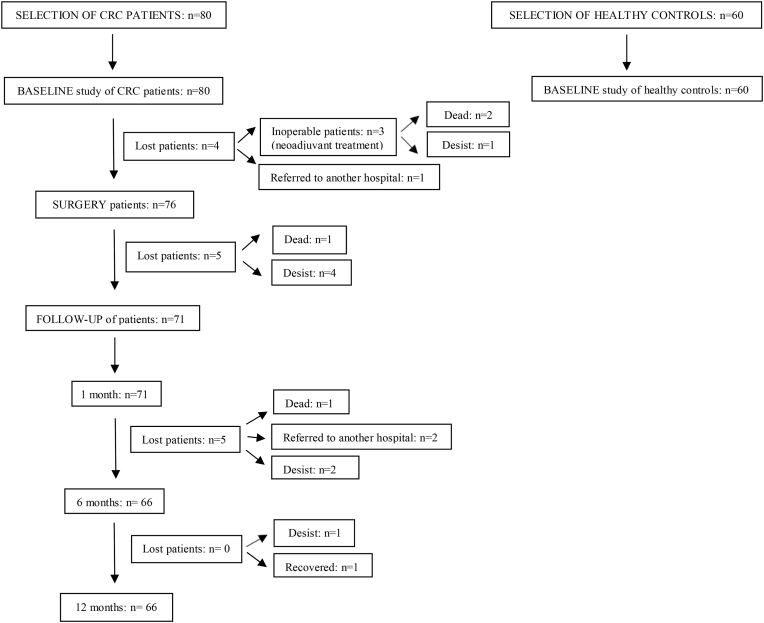
Flow chart of the CRC patients and healthy controls in the longitudinal study.

This was a longitudinal, observational and prospective study involving 80 CRC patients with indication for tumor resection surgery and/or chemoradiotherapy, and 60 healthy controls. Subsequently, we analyzed tumor-related factors and the evolution of the different markers at 1, 6 and 12 months after treatment. The flow chart of the longitudinal study is shown in Fig. 1. Informed consent was obtained from all subjects involved in the study. This study was approved by the Clinical Research Ethics Committee of our hospital and was designed in accordance with the ethical principles of the Declaration of Helsinki (Finland, 1964).

## 3 Methods

Analysis of biochemical parameters, CRP and CEA was performed in an automated chain of analyzers Architect C16000 from Abbott (Chicago, IL, USA). The CA 19.9 and IL-6 were determined by electrochemiluminescence in a Cobas 6000 from Roche Diagnostics (Mannheim, Germany). The white cells and platelets were determined in EDTA-K3 tubes with a Beckman-Coulter LH 500 hematology analyzer (Brea, CA, USA). For fibrinogen analysis, samples of whole blood were assessed with an ACL-TOP of Instrumentation Laboratory Company (Bedford, MA, USA). We analyzed CAT, GSH, oxidized glutathione (GSSG), GSSG/GSH ratio in serum; 8-oxo-7,8-dihidro-2′-deoxiguanosina (8-oxodG) and F2-Isoprotanes (F2-IsoPs) in urine. For determination of OS markers, Cayman Chemical (Ann Arbor, USA) spectrophotometric assays were used, except for 8-oxodG, which was determined by High Performance Chromatography with Electrochemical Detection (HPLC-EC) [2]. The results were relativized with the creatinine levels in urine to eliminate variability in the concentration of samples.

Statistical analysis was performed with the Statistical Package for the Social Sciences (SPSS), 17.0 for Windows (Chicago, USA). Parametric and nonparametric tests were applied to ensure normality of variables and homogeneity of variances.

The results of the controls and CRC groups were compared using the Student's *t*-test. In addition, we analyzed the evolution of different analytes over time with a one-factor ANOVA with repeated measures and Student–Newman–Keuls (SNK) post hoc test to study the differences in the results of the CRC patients between different times. ROC curves were used to study the diagnostic characteristics of inflammatory, tumor and OS markers. Results were considered statistically significant where the two-tailed p value was less than 0.05.

## 4 Results

At baseline, significant differences in age, weight and height, and consequently in body mass index (BMI) were observed between control and CRC groups (Table 1). Therefore, a univariate analysis of variance was performed with BMI and age as covariates, in order to eliminate their possible confounding effect. CRC patients displayed higher levels of glucose, albumin, and transferrin, and lower total cholesterol, HDL and LDL cholesterol, ferritin, iron, transferrin saturation index, hemoglobin and hematocrit than controls. Regarding the presence of comorbidities, the most prevalent associated pathology was dyslipidemia, present in 41 patients (51.3%), followed by hypertension in 39 patients (48.8%), type 2 diabetes mellitus in 32 patients (40%), and obesity in 29 patients (36.3%). Chronic renal failure was found in 12 patients (15%), ischemic heart disease in 6 patients (7.5%), and inflammatory bowel disease in 2 patients (2.5%).

**Table 1:** Anthropometric and biochemical variables of controls and CRC patients at baseline and after 12 months.

Variable	Control (n = 60)	CRC baseline (n = 66)	CRC 12 months (n = 66)	*Adjusted *p*-value	**p-value
Age (years)	64.0 ± 9.0	68.0 ± 11.8	–	0.036	–
Male/Female (n; %)	36/24; 60/40	43/23; 65/35	–	0.550	**-**
BMI (kg/m^2^)	26.1 ± 3.0	28.1 ± 3.9	–	0.001	–
Glucose (mg/dL)	96.2 ± 14.4	117.2 ± 32.8	120.9 ± 34.9	0.001	0.363
Creatinine (mg/dL)	0.9 ± 0.2	0.96 ± 0.31	1.01 ± 0.33	0.269	0.008
Urea (mg/dL)	40.9 ± 7.2	39.5 ± 14.4	42.2 ± 13.8	0.296	0.113
EGF (mL/min)	81.1 ± 8.7	78.6 ± 19.8	73.6 ± 19.8	0.720	<0.001
Total cholesterol (mg/dL)	195.7 ± 34.3	180.0 ± 39.6	187.6 ± 37.7	0.026	0.090
HDL cholesterol (mg/dL)	50.7 ± 12.8	43.7 ± 11.2	48.4 ± 12.9	<0.001	<0.001
LDL cholesterol (mg/dL)	144.9 ± 30.0	114.9 ± 35.1	112.9 ± 35.7	<0.001	0.630
Triglycerides (mg/dL)	112.0 (98.0; 142.8)	108.5 (83.3; 141.0)	110.0 (89.0; 141.3)	0.777	0.189
Uric acid (mg/dL)	4.5 ± 1.6	5.5 ± 1.6	5.8 ± 1.6	0.058	0.006
Albumin (g/dL)	3.9 ± 0.4	4.2 ± 0.5	4.3 ± 0.3	<0.001	0.294
Total proteins (g/dL)	7.0 ± 0.5	6.9 ± 0.5	6.9 ± 0.5	0.577	0.472
Ferritin (μg/L)	133.5 ± 75.4	50.5 ± 65.4	81.1 ± 115.3	0.008	0.042
Iron (μg/dL)	79.7 ± 19.1	61.2 ± 42.5	83.8 ± 32.4	<0.001	<0.001
Transferrin (mg/dL)	269.3 ± 46.5	288.9 ± 49.8	278.4 ± 60.9	0.016	0.133
TSI (%)	30.5 ± 8.9	17.2 ± 11.4	25.1 ± 10.6	<0.001	<0.001
Hemoglobin (g/dL)	14.2 ± 1.5	12.4 ± 1.8	13.5 ± 1.6	<0.001	<0.001
Hematocrit (%)	42.5 ± 4.6	38.2 ± 4.8	41.0 ± 4.7	<0.001	<0.001

* *p*-value adjusted for age and body mass index (BMI) when compared controls vs CRC at baseline; ** *p*-value when compared baseline vs 12 months CRC patients; n: number of cases; EGF: estimated glomerular filtration; TSI: transferrin saturation index. Data are expressed as mean ± standard deviation. In the case of values that did not follow a normal distribution (triglycerides), the median (quartile 25/75) was used.

**Fig. 2:**
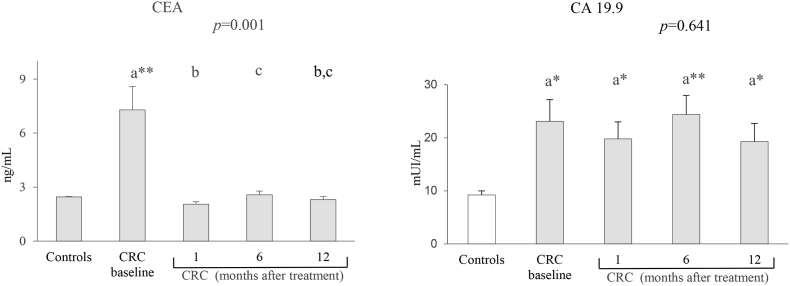
Levels of tumor markers in controls and CRC patients. CEA: carcinoembryonic antigen; CA 19.9: carbohydrate antigen 19.9. Results expressed as mean ± standard error. The statistical differences when comparing patients follow-up times are indicated by the *p* in the upper margin and between each patient are indicated by letters, so that the means sharing these letters do not present statistically significant differences (*p* > 0.05). Differences between the control group and the different patient times are represented by *(*p* < 0.05) and **(*p* < 0.001).

**Fig. 3:**
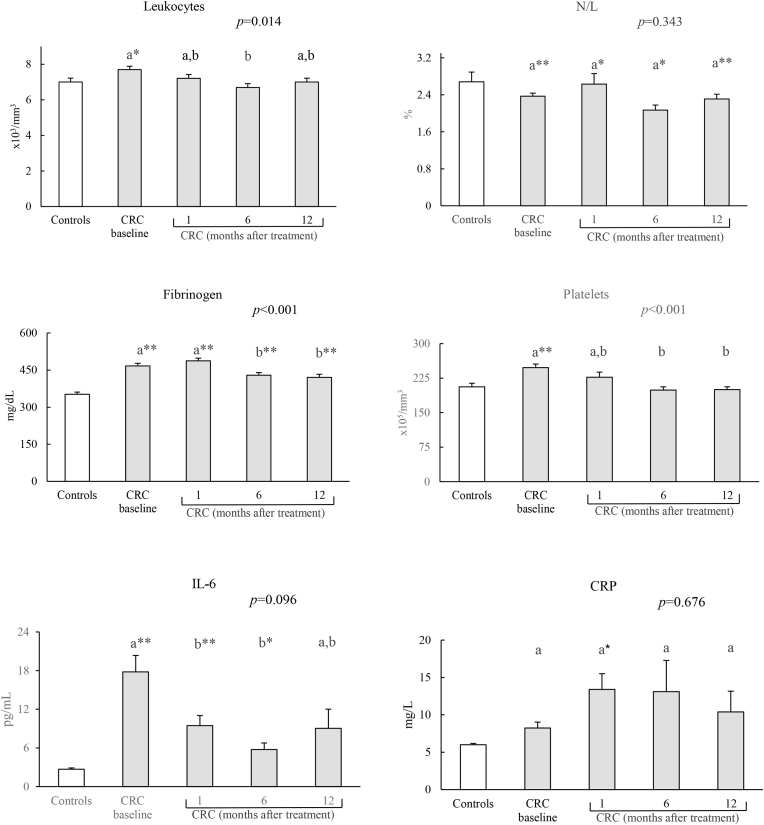
Levels of inflammatory markers in controls and CRC patients. N/L: neutrophil/lymphocyte index; IL-6: interleukin 6; CRP: C-reactive protein. Results expressed as mean ± standard error. The statistical differences when comparing patients follow-up times are indicated by the *p* in the upper margin and between each patient are indicated by letters, so that the means sharing these letters do not present statistically significant differences (*p* > 0.05). Differences between the control group and the different patient times are represented by *(*p* < 0.05) and **(*p* < 0.001).

**Fig. 4:**
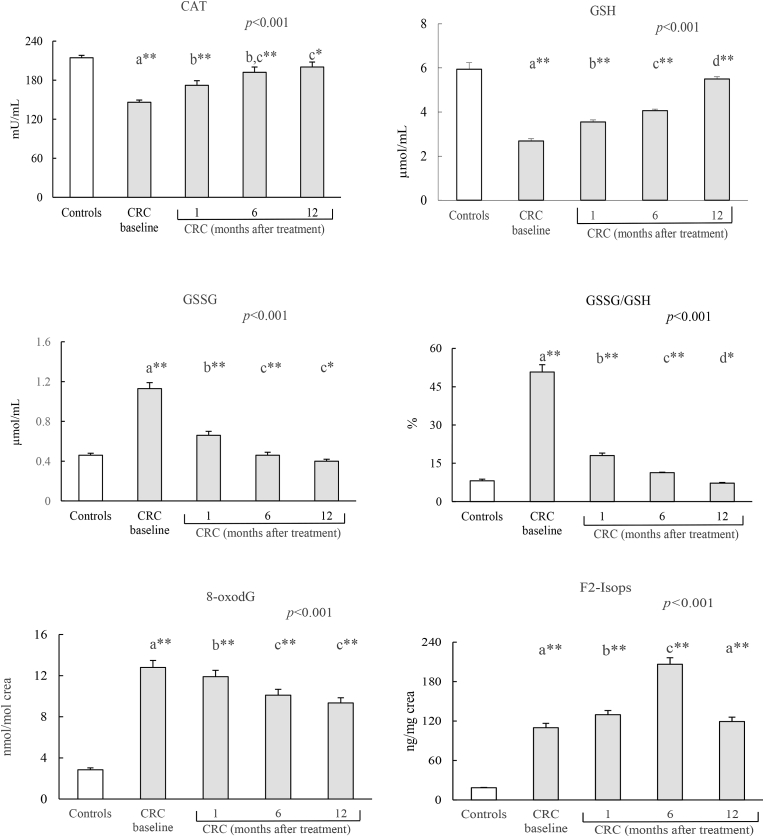
Levels of oxidative stress markers in controls and CRC patients. CAT: catalase; GSH: reduced glutathione; GSSG: oxidized glutathione; 8-oxodG: 8-oxo-7,8-dihydro 2′-deoxy-guanosine; F2-IsoPs: F2-Isoprostanes. Results expressed as mean ± standard error. The statistical differences when comparing patients follow-up times are indicated by the *p* in the upper margin and between each patient are indicated by letters, so that the means sharing these letters do not present statistically significant differences (*p* > 0.05). Differences between the control group and the different patient times are represented by *(*p* < 0.05) and **(*p* < 0.001).

As expected, levels of CEA and CA 19.9 tumor and inflammatory markers were significantly higher in CRC patients than in controls at baseline (Fig. 2, Fig. 3). In the same way, the mean of OS markers showed significant differences between the control group and CRC patients after adjustment for age and BMI covariates (Fig. 4).

In the post-treatment follow-up, a total of 66 CRC patients were analyzed, of which 18 (27.3%) showed poor response to treatment, and 4 of them died. Table 2 shows the cut-off of the different markers from which it is considered positive and the percentage of sensitivity at baseline and after follow-up of cases with worse prognosis. Tumor and inflammatory markers showed a low sensitivity with values below 40%. On the other hand, from the OS markers, the glutathione system detected approximately >65% of the cases, while CAT and especially 8-oxodG and F2-IsoPs detected practically all the cases. Receiver operating characteristic (ROC) curves for the tumor, OS and inflammatory markers are shown in Supplementary Fig. 1. Antioxidant markers (CAT and GSH) are presented separately because they are considered positive below the cutoff point. In addition, OS markers were studied according to clinical markers of prognosis (Table 3). It is worth noting that significant differences were found in relation to histological tumor grade (GSSG/GSH index and 8-oxodG), post-surgery treatment (CAT and GSH), location in the right or left colon (GSH and 8-oxodG), occurrence of post-surgery recurrences and synchronous tumors with GSH and coexistence of diverticulosis and occurrence of post-surgery metastasis with F2-IsoPs.

**Table 2:** Sensitivity of the OS and inflammatory markers at baseline and after 12-month follow-up.

Marker	Cut-off (positive result)	Baseline sensitivity (%) n = 80	Poor evolution sensitivity (%) n = 18
CEA (ng/mL)	>5	26.3	44.4
CA 19.9 (UI/mL)	>40	17.5	50
IL-6 (pg/mL)	>7	55	44.4
CRP (mg/L)	>10	41.3	38.9
Leukocytes (x10^3^/mm^3^)	>11.3	3.8	22.2
N/L (−)	>5	2.5	0
Platelets (x10^5^/mm^3^)	>450	1.3	0
Fibrinogen (mg/dL)	>500	37.5	55.5
CAT (mU/mL)	<191	80	100
GSH (μmol/mL)	<3.17	78.8	66.6
GSSG (μmol/mL)	>0.73	75	83.3
GSSG/GSH (%)	>14.3	98.8	72.2
8-oxodG (nmol/mmol crea)	>5.87	92.5	94.4
F2-IsoPs (pg/mg crea)	>94.5	78.8	77.8

**Table 3:** Levels of OS markers in serum and urine in CRC patients according to tumor-related factors.

	CAT (mU/mL)	GSH (μmol/mL)	GSSG (μmol/mL)	GSSG/GSH (%)	8-oxodG (nmol/mmol crea)	F2-IsoPs (pg/mg crea)
**History of CRC**						
No	154.2 ± 57.2	2.45 ± 0.73	1.13 ± 0.45	51.6 ± 29.1	12.7 ± 4.60	109.1 ± 57.4
Yes	141.0 ± 45.3	2.36 ± 0.64	0.99 ± 0.32	45.2 ± 21.1	12.6 ± 5.09	109.4 ± 53.3
p value	0.349	0.620	0.223	0.244	0.917	0.943
**WHO histological grade**						
Low	152.3 ± 51.3	2.46 ± 0.70	1.06 ± 0.43	48.2 ± 29.8	12.2 ± 4.54	108.8 ± 55.9
High	115.6 ± 39.8	2.12 ± 0.63	1.43 ± 0.60	72.7 ± 36.6	16.9 ± 4.70	114.2 ± 59.3
p value	0.072	0.152	0.086	**0.021**	**0.003**	0.341
**Tumor diameter**						
<6 cm	155.1 ± 54.3	2.40 ± 0.69	1.05 ± 0.45	48.9 ± 29.9	12.6 ± 4.61	108.9 ± 56.7
≥6 cm	121.0 ± 48.3	2.47 ± 0.76	1.33 ± 0.50	62.4 ± 40.3	12.4 ± 4.74	101.2 ± 55.4
p value	0.072	0.787	0.092	0.230	0.919	0.580
**Pre-surgery treatment**						
No	149.6 ± 57.2	2.39 ± 0.66	1.08 ± 0.48	50.3 ± 31.3	12.7 ± 5.05	108.9 ± 57.3
Yes	150.3 ± 39.6	2.54 ± 0.96	1.31 ± 0.40	60.1 ± 34.2	13.5 ± 2.91	100.3 ± 59.2
p value	0.970	0.614	0.129	0.345	0.597	0.340
**Post-surgery treatment**						
No	161.5 ± 53.1	2.61 ± 0.76	1.04 ± 0.46	46.1 ± 32.3	12.0 ± 4.35	105.9 ± 18.3
Yes	137.8 ± 48.4	2.19 ± 0.56	1.14 ± 0.46	55.7 ± 29.0	13.1 ± 4.92	112.5 ± 13.0
p value	**0.040**	**0.008**	0.369	0.182	0.327	0.076
**Tumor localization**						
Right	149.0 ± 52.8	2.52 ± 0.76	1.06 ± 0.48	48.6 ± 33.1	11.6 ± 4.20	110.4 ± 57.8
Left	143.3 ± 55.4	2.13 ± 0.48	1.09 ± 0.50	54.4 ± 30.9	14.5 ± 7.76	111.0 ± 57.4
p value	0.682	**0.039**	0.812	0.519	**0.044**	0.336
**Complications**^a^						
No	153.3 ± 54.4	2.44 ± 0.72	1.08 ± 0.47	50.0 ± 31.9	13.0 ± 4.86	108.6 ± 16.9
Yes	141.6 ± 48.4	2.23 ± 0.53	1.12 ± 0.40	53.8 ± 27.3	10.8 ± 3.07	110.7 ± 13.7
p value	0.471	0.222	0.760	0.667	0.108	0.663
**Tumor recurrence**						
No	151.3 ± 53.1	2.43 ± 0.71	1.08 ± 0.44	49.7 ± 30.2	12.5 ± 4.63	109.1 ± 56.1
Yes	155.4 ± 82.7	1.82 ± 0.99	1.42 ± 0.92	79.5 ± 54.9	14.5 ± 3.38	109.8 ± 65.8
p value	0.928	**0.002**	0.298	0.178	0.554	0.983
**Presence of diverticulosis**						
No	150.8 ± 55.4	2.36 ± 0.69	1.12 ± 0.48	52.3 ± 31.1	12.8 ± 4.74	101.7 ± 59.0
Yes	149.0 ± 52.5	2.53 ± 0.77	1.04 ± 0.46	47.9 ± 34.2	12.5 ± 5.06	111.4 ± 55.3
p value	0.935	0.447	0.566	0.633	0.814	**0.040**
**Post-surgery metastasis**						
No	154.4 ± 52.2	2.45 ± 0.70	1.04 ± 0.41	42.4 ± 27.5	12.2 ± 4.17	106.9 ± 56.1
Yes	131.9 ± 58.3	2.21 ± 0.77	1.44 ± 0.61	65.2 ± 42.5	15.1 ± 6.54	123.2 ± 67.0
p value	0.200	0.326	0.072	0.086	0.199	**<0.001**
**Synchronous tumors**						
No	147.6 ± 54.3	3.08 ± 0.68	1.12 ± 0.48	53.0 ± 31.9	13.0 ± 4.87	110.1 ± 56.4
Yes	184.1 ± 43.4	2.36 ± 0.67	0.87 ± 0.18	30.2 ± 10.9	10.9 ± 2.75	104.5 ± 54.7
p value	0.104	**0.013**	0.213	0.087	0.302	0.415
**Co-existence of adenomas**						
No	146.3 ± 55.2	2.44 ± 0.66	1.06 ± 0.44	47.3 ± 27.6	12.6 ± 4.72	110.1 ± 55.5
Yes	154.9 ± 54.0	2.36 ± 0.75	1.16 ± .050	56.4 ± 35.5	12.9 ± 4.90	108.9 ± 60.6
p value	0.488	0.591	0.357	0.201	0.848	0.754

a Include post-operative complications (ileus, bleeding, suture dehiscence and occlusions).

In reference to the tumor markers, the average value of CEA in the three monitoring times (1, 6 and 12 months) was similar to the value of the controls, without significant differences between them, unlike CA 19.9, which showed significant differences in all of them, but with mean results similar to the baseline (Fig. 2).

In relation to the inflammatory markers, a significant decrease was found in the monitoring of these markers, except for CRP (Fig. 3).

All the OS markers of the CRC patients presented significant differences during the follow-up with respect to controls, especially at baseline and at 1 and 6 months (p < 0.001); at 12 months the differences were less significant for CAT, GSSG and GSSG/GSH (p < 0.05) (Fig. 4). Thus, OS markers tended to progressively recover 12 months after treatment, with values close to those of the control group.

## 5 Discussion

OS and inflammation are known to play an important role in CRC. Moreover, the identification of easily determined biochemical molecules for their use as clinical markers of diseases continues to be a topic of great interest in translational research, especially when cancer is considered. This fact is especially important in the case of gastrointestinal tumors, where there is a need to have sufficiently reproducible, sensitive, and specific markers that meet clinical expectations.

We have characterized the systemic OS in CRC patients by analyzing the antioxidant enzyme activities, the redox state and the degree of DNA damage. As compared with healthy subjects, an important increase of the OS status has been observed in the CRC group. Moreover, the surgical curative and locoregional interventions in those patients tend to progressively recover the control levels of antioxidant enzyme activities and to decline oxidative byproducts both in serum or urine after 12 months of treatment. In addition, significant differences were found in OS markers according to related-tumor factors such as histological tumor grade, location in the right or left colon, synchronous tumors and post-surgery treatment, metastasis and tumor recurrences.

Tumor markers have been used for a long time as evolutionary control of CRC, rather than as diagnosis due to their lack of sensitivity. In fact, and according to the American Society of Clinical Oncology and the European Society of Medical Oncology guidelines, CEA is not recommended for use in screening because it is rarely elevated in CRC stage I [14,15]. Regarding CA 19.9, most studies conclude that CA 19.9 is much less sensitive than CEA [16] and that elevated CA 19.9 levels are considered a poor prognostic factor, as it occurs with CEA [17,18].

Cancer progression and survival are not determined only according to the local tumor characteristics, but also the host inflammatory response. This response showed increased blood levels of white blood immune cells, neutrophils, the neutrophil-to-lymphocyte ratio (N/L), platelets, fibrinogen, CRP and cytokines such as IL-6. A systemic inflammatory response is consistently associated with a poor outcome, independently of the tumor stage [19,20]. In general, the most common proinflammatory systemic effects are leukocytosis, neutrophilia and lymphopenia. These hematological findings correlated significantly with the advanced stage of the tumor and therefore with a poor prognosis of the disease [21]. All markers of inflammation in our CRC patients showed significant differences with healthy controls and improved after treatment.

According to other studies [9,11], the comparison of means between the patients versus the group of healthy controls showed highly significant differences in all the OS markers studied. The means of CAT and GSH were lower and the rest of markers were higher in patients than in controls.

In general, any condition associated with excess ROS can decrease tissue immunoexpression of CAT [22] and serum levels of CAT and GSH [11,23]. Baltruskeviciene et al., in a study of 40 healthy controls and 58 CRC patients, found that serum GSH was significantly lower in patients than in controls, with similar results to those obtained in our study [24]. Also, Dusak et al. reported decreased levels of GSH in serum of 25 CRC patients with respect to healthy controls [9].

Another OS marker that increases in different types of cancer is 8-oxodG in urine [25,26]. Roszkowski et al. determined 8-oxodG in plasma, serum and leukocytes of patients with CRC, and they found higher levels than in controls [27]. In addition, the measurement of 8-oxodG in urine compared to tissue or lymphocytes had the advantage of being a non-invasive method that remains stable at −20 °C for a long period of time.

F2-IsoPs are considered the most reliable markers to monitor OS in vivo due to their high chemical stability and sensitivity to changes in OS, according to some authors [28]. Forman et al. [29] discussed that F2-IsoPs are the best markers of lipid peroxidation that currently exist. In a study of 50 CRC patients and 20 controls, Rasool et al. found significantly higher levels of F2-IsoPs in patients with CRC than in controls, together with low levels of CAT and GSH, in agreement with our results [30]. For Il'yasova et al., the recommended biomarkers to monitor oxidative status over time were 8-oxodG and F2-IsoPs [31], and different authors also combine both for the study of OS [32,33].

On the other hand, we did not find other studies that assess postoperative monitoring of OS markers in patients with CRC. In gastric cancer, Borrego et al. [25] followed up 48 patients for 12 months and determined OS markers in tumor, peripheral mononuclear cells and 8-oxodG in urine; the patients exhibited elevated levels of lipid peroxidation and DNA damage with increased malondialdehyde (MDA) and 8-oxodG, which progressively decreased after surgery to levels close to those of healthy individuals at 12 months.

In agreement with our study, Farias et al. correlated the levels of lipid peroxidation and other OS markers with the evolution of CRC in 43 patients, and found that lipid peroxidation markers increase before other biomarkers, and therefore could be useful in the prognosis of CRC. In fact, they observed increases in CAT levels of patients with a good evolution after chemotherapy treatment [34]. In contrast, Gopčević et al., in a study that included 70 CRC patients and 42 healthy controls, found higher CAT levels in the CRC patient group [35].

In our study, all the OS markers of the CRC patients presented significant differences during the follow-up compared with the control group, especially at baseline and at 1 and 6 months; at 12 months, the differences were less significant for CAT, GSSG and GSSG/GSH.

The evolution of OS markers over time was, in general, progressive; lower basal levels were found in patients compared to controls in the case of the antioxidants CAT and GSH and, on the contrary, higher levels in the rest of the pro-oxidant markers. This effect is observed in those patients undergoing efficient treatment. At 12 months after treatment, low levels of CAT and GSH increased and almost reached control group levels, while 8-oxodG and F2-IsoPs remained high with respect to control values. With regard to the detection of CRC patients who progressed poorly after surgical treatment, CA 19.9 improved the sensitivity with respect to CEA, but both were below 40%. Inflammatory markers were also insensitive in their detection, especially CRP and white series; fibrinogen was the most sensitive of them all. On the other hand, from the OS markers, the glutathione system detected approximately 50% of the cases, while CAT and especially 8-oxodG and F2-IsoPs detected practically all the cases with worse prognosis.

One of the strengths of this study is that OS markers were used not only to compare differences with controls and CRC groups, but also to monitor the disease progression over a one-year time period after surgical treatment. On the other hand, we should point out some limitations, as OS markers have not been evaluated in other gastrointestinal diseases and their efficiency as a clinical tumor marker should be studied more extensively.

## 6 Conclusions

The present study provides evidence that a significant increase of the OS status was observed in the CRC patients when compared with healthy controls, with a decrease in the antioxidant markers CAT and GSH and an increase in the pro-oxidant markers GSSG, 8-oxodG and F2-IsoPs, suggesting a strong relationship between OS and CRC. After treatment, CRC patients tend to progressively recover to control levels.

Based on the obtained results and the correlation analysis, we propose the assay of urinary 8-oxodG and F2-IsoPs, as well as serum CAT, GSH, GSSG as valid markers for OS evaluation and the clinical follow-up of CRC patients.

## Funding

This work was partially supported by grants UGP-19-037 and UGP-20-132 from FISABIO, and PI21/01160 from 10.13039/501100004587Instituto de Salud Carlos III, and was co-funded by the European Regional Development Fund (ERDF “A way to build Europe”). C.B. is a recipient of a Miguel Servet contract (CP19/00077) from the 10.13039/501100004587Instituto de Salud Carlos III.

## Declaration of competing interest

None.

## Data Availability

Data will be made available on request.

## Appendix A Supplementary data

Supplementary Fig. 1 Receiver operating characteristic (ROC) curves for the analyzed markers. A: Catalase (CAT) and reduced glutathione (GSH). B: CEA, CA 19.9, interleukin 6 (IL6), C-reactive protein (CRP), leukocytes, neutrophil/lymphocyte (N/L) index, platelets, fibrinogen, oxidized glutathione (GSSG), GSSG/GSH ratio, 8-oxo-7,8-dihydro-2′-deoxyguanosine (8-oxodG) and F2-IsoProstanes (F2-IsoPs). C. Area under the curve and 95% confidence interval. Supplementary Fig. 1
