# Supplementary material for: The role of oxidative stress, tumor and inflammatory markers in colorectal cancer patients: A one-year follow-up study
Source: Redox Biol. 2023 Mar 8;62:102662. doi: 10.1016/j.redox.2023.102662 (PMC10023975; doi:10.1016/j.redox.2023.102662)
Supplement: Supplementary Fig. 1 — Receiver operating characteristic (ROC) curves for the analyzed markers. A: Catalase (CAT) and reduced glutathione (GSH). B: CEA, CA 19.9, interleukin 6 (IL6), C-reactive protein (CRP), leukocytes, neutrophil/lymphocyte (N/L) index, platelets, fibrinogen, oxidized glutathione (GSSG), GSSG/GSH ratio, 8-oxo-7,8-dihydro-2′-deoxyguanosine (8-oxodG) and F2-IsoProstanes (F2-IsoPs). C. Area under the curve and 95% confidence interval. [file mmc1.pdf]

A. ROC curve: antioxidant markers

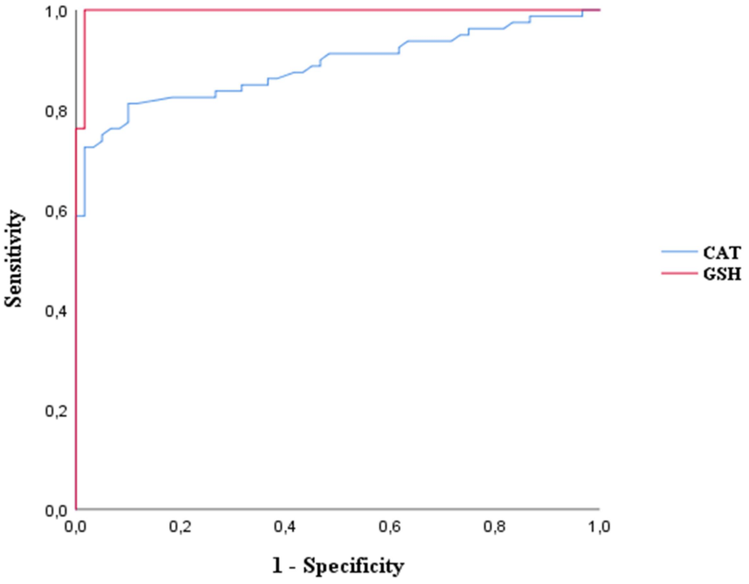

B. ROC curve: oxidative stress, inflammatory and tumor markers

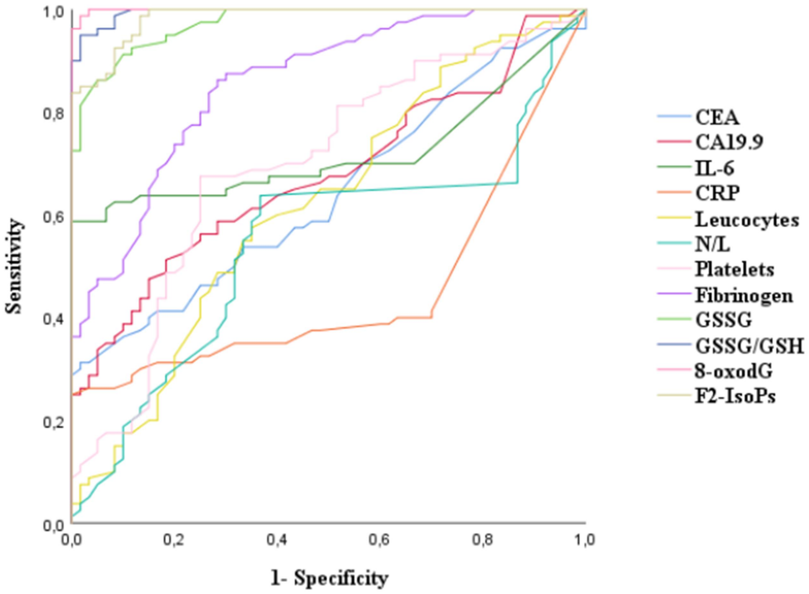

C. Area under the curve and 95% confidence interval

Marker	Area under the curve (AUC)	95% asymptotic confidence interval	
		Lower limit	Upper limit
CAT	0.888	0.832	0.943
GSH	0.997	0.990	1.003
GSSG	0.974	0.954	0.994
GSSG/GSH	0.995	0.989	1.002
8-oxodG	0.999	0.997	1.000
F2-IsoPs	0.997	0.990	1.003
CEA	0.642	0.552	0.732
CA 19.9	0.677	0.590	0.765
IL-6	0.721	0.633	0.809
CRP	0.450	0.351	0.548
Leucocytes	0.961	0.525	0.714
N/L	0.650	0.546	0.754
Platelets	0.695	0.606	0.784
Fibrinogen	0.856	0.796	0.917
